# Prevalence of different comorbidities in COPD patients by gender and GOLD stage

**DOI:** 10.1186/s40248-015-0023-2

**Published:** 2015-08-05

**Authors:** R. W. Dal Negro, L. Bonadiman, P. Turco

**Affiliations:** National Centre for Respiratory Pharmacoeconomics and Pharmacoepidemiology, CESFAR, Verona, Italy; CEMS, Specialist Medical Centre, Verona, Italy; Research & Clinical Governance, Verona, Italy

**Keywords:** Comorbidities, COPD, Gender, GOLD severity

## Abstract

**Background:**

Several comorbidities frequently affect COPD progression. **Aim** of the study was to assess the prevalence of main comorbidities by gender and disease severity in a cohort of COPD patients referring for the first time to a specialist institution.

**Methods:**

The study was a non-interventional, cross-sectional investigation carried out via automatic and anonymous selection from the institutional data base over the period 2012–2015. Inclusion criteria were: subjects of both sex aged ≥40 years; diagnosis of COPD according to GOLD guidelines 2014; the availability of a complete clinical record file. Variables collected were: lung function; smoking history; BMI; the Charlson Comorbidity Index (CCI); number and kind of comorbidities for each patient.

**Results:**

At least one comorbidity of clinical relevance was found in 78.6 % of patients, but at least two in 68.8 %, and three or more were found in 47.9 % of subjects. Mean CCI was 3.4 ± 1.6sd. The overall prevalence was 2.6 comorbidities per patient, but 2.5 in males, and 3.0 in females, respectively (*p* < 0.05). Cardio-vascular disorders were the most frequent, but significantly more frequent in males (44.7 vs 30.7 %, respectively), while the metabolic, the digestive and the osteo-articular disorders were prevailing in females (12.4 vs 9.2; 14.2 vs 4.8, and 6.0 vs 3.8, respectively). In particular, chronic cor pumonale and arrhythmias mainly prevailed in men and congestive heart failure in females, while arterial hypertension resulted equally distributed. As concerning respiratory disorders, pneumonia, pleural effusions and chronic respiratory failure were more frequently found in men, while bronchiectasis and asthma-COPD overlap syndrome (ACOS) in females. Anaemia, gall bladder stones, osteoporosis and spontaneous fractures mostly prevailed in females, while gastric disorders of inflammatory origin and arthrosis were more frequent in males. Cognition disorders, dementia and signs of degenerative brain disorders were more frequently found in men, while depression in females. Finally, lung cancer was at the first place in men, but at the second in females.

**Conclusions:**

All comorbidities increased their prevalence progressively up to the last stage of COPD severity, except the cardio-vascular and the metabolic ones which dropped in the IV GOLD stage, presumably due to the high mortality rate in this severe COPD stage. The gender-dependency of comorbidities was confirmed in general terms, even if lung cancer proved a dramatic increase almost independently of sex.

## Background

Chronic obstructive pulmonary disease (COPD) is the chronic respiratory condition that currently represents the most significant health problem at international level [1]. The epidemiological, clinical and socio-economic impact of COPD still is constantly increasing, and COPD is projected to be the 3^rd^ leading cause of death in the world by 2030, and the 7^th^ as a burden of disease [2, 3].

COPD can progressively affect the function of other organs (e.g. heart, vasculature, muscles, kidney, liver, gastro-enteric apparatus, and brain); it is frequently associated with various disorders [4–7] and presumed to accelerate lung ageing [8, 9]. Several comorbidities frequently prevail particularly in elderly patients, but the relationship linking their prevalence to patients’ gender and COPD severity is still debated [10–12].

Aim of the study was to investigate in real life the prevalence of different comorbidities by gender and disease severity by GOLD stage in a cohort of COPD patients referring to a specialized institution for the first time.

## Methods

The study was a non-interventional, cross-sectional investigation carried out on the centralized Data Base of the Lung Unit. Data of COPD subjects sequentially referring for the first time to the specialist centre over the period May 2012 - April 2015 were automatically and anonymously selected [13]. Inclusion criteria were: 1) males or females aged ≥ 40 years; 2) diagnosis of COPD according to the GOLD guidelines 2014 [1]; 3) availability of a complete clinical file, including the patients’ history, with the specific comorbidity section properly filled; 4) availability of a complete lung function.

Variables collected in all subjects were: age; gender; smoking history; the Body-Mass Index (BMI); the Charlson Comorbidity Index (CCI); the post-bronchodilator (salbutamol 400mcg) Forced Expiratory Volume in one second (FEV_1_) reported as absolute value in litres, and as % predicted value; and the FEV_1_/FVC (Forced Vital Capacity) predicted % ratio.

Comorbidities were firstly grouped into the following groups: cardio-vascular; respiratory; metabolic; oncologic; digestive; neurologic/ psychiatric; osteo-articular disorders. In a second phase, the most frequent disorders reported in each group were analytically ranked, and their distribution compared by gender. Finally, all comorbidities were ranked by groups according to the different GOLD stages [1].

### Statistics

Descriptive and non-parametric test; *p* < 0.05 was assumed as the lowest level for statistical significance.

## Results

Patients’ files fitting with the inclusion criteria were 1,216; males were *n* = 880 (72.4 %), and females *n* = 336 (27.6 %). The basic characteristics of the two patients’ groups proved well matched and are reported in Table 1. Active smokers were slightly prevailing in females, while quitters in males.

**Table 1:** Basic characteristics of the cohort

	Males (*n* = 880)	Females (*n* = 336)	
			*P*
Mean age (y)	70.6 ± 9.9	69.7 ± 10.2	ns
Current smoker (n)	218 (24.8 %)	91 (27.1 %)	
Ex smoker (n)	516 (58.6 %)	192 (57.1 %)	
BMI	28.6 ± 6.8	27.6 ± 5.5	ns
FEV_1_ (% pred.)	61.0 ± 19.8	63.7 ± 22.9	ns
FEV_1_ (L)	1.4 ± 0.4	1.2 ± 0.7	ns
FEV_1_/FVC	55.2 ± 9.8	56.3 ± 9.1	ns
C C I	3.5 ± 1.9	3.4 ± 2.2	ns

A total of 3,198 comorbidities were recorded. At least one comorbidity of clinical relevance was recorded in 78.6 % of subjects, while at least two were found in 68.8 % of subjects, and three or more in 47.9 %. Their mean prevalence was 2.6 per patient, and the mean CCI was 3.4 ± 1.6. Moreover, comorbidities amounted to 2,182 in males and to 1,016 in females, with a corresponding mean prevalence of 2.5 and of 3.0 per patient, respectively (*p* < 0.05).

In particular, cardio-vascular disorders were the most represented (40.0 %), while the prevalence of respiratory and metabolic disorders was 24 % and 11.4 %, respectively. Finally, the prevalence of digestive; oncologic, neurologic/psychiatric, and osteo-articular disorders was 7.8 %; 7.5 %; 5.4 %, and 3.9 %, respectively.

Cardio-vascular comorbidities were significantly more frequent in males (44.7 % vs 30.7 %, respectively), while metabolic, digestive, and osteo-articular disorders prevailed in females (12.4 % vs 9.2 %; 14.2 % vs 4.8 %; 6.0 % vs 3.8 %, respectively). Respiratory, oncologic, and neuro-psychiatric disorders resulted equally distributed in both sex (Table 2).

**Table 2:** % distribution of different groups of comorbidities in the whole sample and by gender

Comorbidities	Overall % (*n* = 3,198)	Males % (*n* = 2,182)	Females % (*n* = 1,016)
Cardio-Vascular	39.0	44.7	30.7
Respiratory	22.4	23.0	21.1
Metabolic	10.4	9.2	12.4
Oncologic	7.6	7.0	8.5
Neuro Psychiatric	6.5	6.6	6.2
Gastro Enterologic	8.6	4.8	14.2
Osteo-Articular	4.9	3.8	6.0
Other	0.6	0.9	0.9

**Fig. 1:**
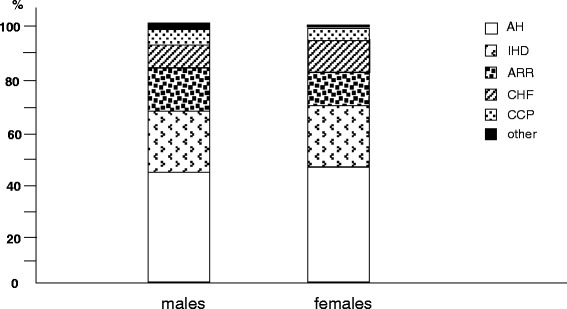
% prevalence of different cardio-vascular disorders by gender: AH, arterial hypertension; IHD, ischemic heart disease; ARR, arrhytmias; CHF, congestive heart failure; CCP, chronic cor pulmonale; other

Among cardio-vascular comorbidities, arterial hypertension (AH) and ischemic heart diseases (IHD) were the most frequent ones, and proved equally distributed in both sex. On the contrary, arrhythmias and chronic cor pulmonale (CCP) prevailed in males, while congestive heart failure (CHF) in females (Fig. 1).

**Fig. 2:**
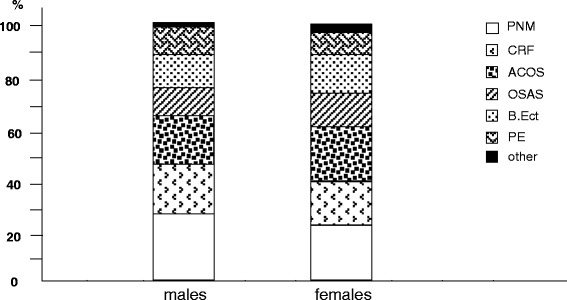
% prevalence of different respiratory disorders by gender: PNE, pneumonia; CRF, chronic respiratory failure; ACOS, asthma-Chronic obstructive pulmonary disease overlap syndrome; OSAS, obstructive sleep apnoea syndrome; B.Ect., bronchiectasys; PL, pleural effusion; other

As concerning respiratory disorders, pneumonia; chronic respiratory failure (CRF), and pleural effusions were more frequently recorded in males, while bronchiectasys and asthma-COPD overlap syndrome (ACOS) in females (Fig. 2).

**Fig. 3:**
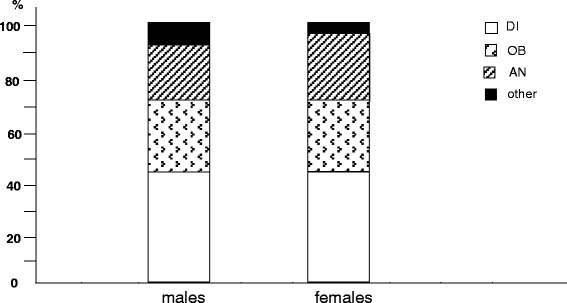
% prevalence of different metabolic disorders by gender: DI, diabetes; OB, obesity; AN, anaemia; other

Anaemia was the metabolic disorder mostly prevailing in females, while obesity and diabetes were homogeneously distributed in both sex (Fig. 3).

**Fig. 4:**
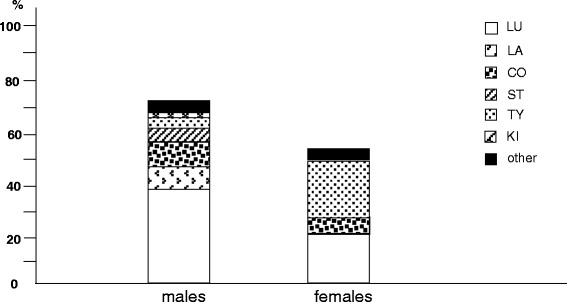
% prevalence of different oncologic disorders by gender: Lu, lung; LA, larynx; CO, colon; ST, stomach; TY, Tyroid; KI, kidney; other

**Fig. 5:**
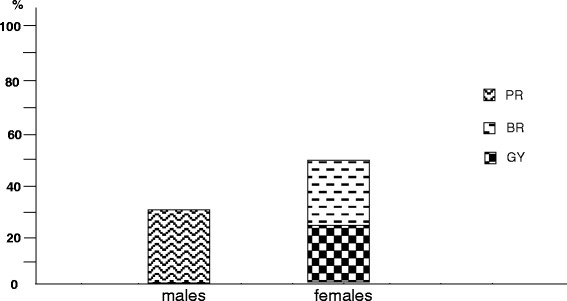
% prevalence of different gender-specific oncologic disorders by gender: PR, prostate; BR, breast; GY, gynaecological district

Lung cancer was the most prevalent oncologic disease in men, followed by the cancer of colon and of larynx. On the other hand, lung cancer was the second most frequent cancer in females, only preceded by the thyroid cancer. Moreover, as concerning the gender-specific oncologic diseases, the prostate cancer in males, and those of the breast and of the gynecological district in females were the most frequent, respectively (Figs. 4 and 5).

**Fig. 6:**
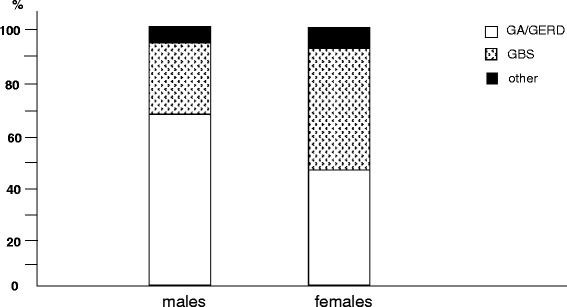
% prevalence of different digestive disorders by gender: GA/GERD, gastritis/gastro-oesophageal reflux; GBS, gall bladder stones; other

Gastric inflammatory disorders (such as, peptic ulcer and troubles due to gastro-oesophageal reflux - GERD) we the most frequent in males, while the prevalence of gall bladder stones was higher in females (Fig. 6).

**Fig. 7:**
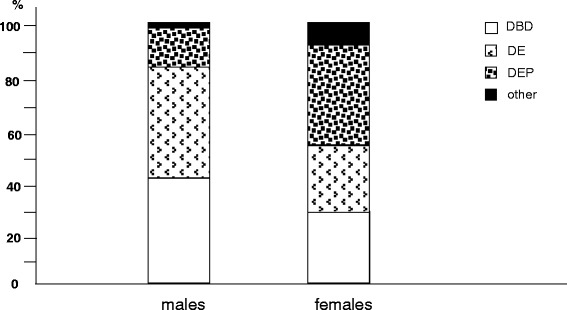
% prevalence of different neurologic/psychiatric disorders by gender: DBD, degenerative brain disorders; DE, dementia; DEP, depression, other

Among the neuro-psychiatric disorders, dementia and signs of degenerative brain disorders were much more frequent in males, while depression proved highly prevalent in females (Fig. 7).

**Fig. 8:**
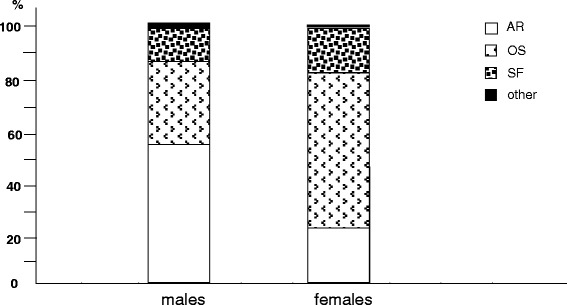
% prevalence of different osteo-articular disorders by gender: AR, arthrosis; OS, osteoporosis; SF, spontaneous fractures; other

Finally, within osteo-articular disorders, arthrosis was mostly reported in males, while osteoporosis and the occurrence of spontaneous fractures in females (Fig. 8).

**Fig. 9:**
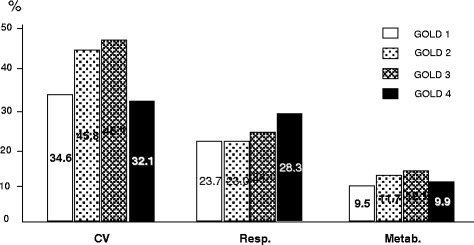
% prevalence of cardio-vascular (CV); respiratory (Resp.), and metabolic (Metab.) comorbidities by GOLD stage of severity

**Fig. 10:**
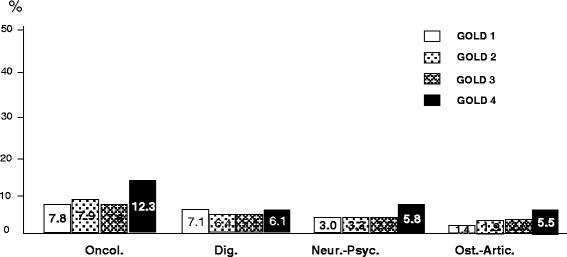
% prevalence of oncologic (Oncol.); digestive (Dig.); neurological/psychiatric (Neur/Psyc), and osteo-articular (Ost.-Artic.) comorbidities by GOLD stage of severity

The prevalence of different groups of comorbidities by the GOLD severity stage is reported in Figs. 9 and 10. In general, except digestive disorders which proved equally distributed within the different levels of COPD severity, all other comorbidities increased their prevalence progressively according to COPD severity. Only metabolic and cardio-vascular comorbidities showed a significant drop of their prevalence just in the IV GOLD stage (*p* < 0.02 an *p* < 0.05, respectively), being this feature particularly clear for the cardio-vascular disorders.

## Discussion

Chronic obstructive pulmonary disease (COPD) is one of the major causes of morbidity and mortality worldwide. The progressive increase of its epidemiological and socio-economic impact is endless, also due to the presence of several comorbidities which can affect substantially the clinical progression of COPD, together with the patients’ quality of life and survival.

In the last decades many studies were particularly aimed to assess and characterize the prevalence of comorbidities in COPD patients, and in the majority of these studies the overall prevalence is confirmed quite high, ranging between 65-81 % of subjects [14–19]. Data of the present study, even if collected according to a different protocol design, are in general agreement with those of a recent Italian survey which found a quite similar rate of comorbidities in comparable COPD subjects [19]. These data further proves that the prevalence of comorbidities is very high also in a cohort of COPD patients referring for the first time to a specialist institution, because 73.8 % of patients aged 70 years had at least one comorbidity of clinical relevance. This figure, when compared to that of a previous study carried out on a quite similar cohort of Italian COPD patients of the same age (70.3 years) [20], is suggesting that an increase of 16.1 % in the general prevalence of comorbidities occurred over less than a decade. The high impact of comorbidities is further emphasized by the evidence that two or more disorders were recorded in the vast majority of patients (68.8 %), and that three or more were also found in near 50 % of COPD subjects. These data confirm those of other recent studies focusing the same aspect in COPD [3, 21, 22].

Actually, the clinical relevance of these figures is also emphasized by the mean CCI value assessed in the present paper, which was higher than that found in other studies [23] (3.4 vs 2.5, respectively), thus contributing to support and explain the higher prevalence of comorbidities per patient found in our cohort of patients (i.e. an average of 2.6). Moreover, the prevalence of comorbidities proved significantly higher in females than in males (3.0 vs 2.5 per patient, respectively), thus showing a clear gender-dependent trend which might support the hypothesis of the heavier global COPD impact in females than in males.

From a general point of view, cardiovascular disorders proved once again the most represented in COPD patients, with a percentage frequency absolutely comparable to that of other recent studies [3, 7–9, 19, 24], even if with some differences, such as, congestive heath failure were most represented in females, and chronic cor pulmonale and arrhythmias in males. Ischemic heart disease were equally distributed in both sex and this result might be related to the ever increasing tobacco use among females in our country during the last decades.

A trend in favour of a gender-dependency was also found in respiratory disorders: differently from pneumonia, chronic respiratory failure and pleural effusions which were more frequent in males, bronchiectasis and ACOS prevailed in females. In particular, the ACOS prevalence assessed in the present study was very similar (i.e. 22 %) to that described in some specific studies which positioned this respiratory complex disorder around the 15–25 % of all COPD phenotypes [3, 25–27].

In the present study, metabolic disorders (in particular, diabetes and obesity) were less represented than in other studies [3] and it might be presumably related to the differences in the Italian alimentary style and the daily diet also in COPD patients. Also anaemia was recognised with a frequency very close to that of other studies [28, 29], even if in the present study anaemia was once again confirmed as significantly more represented in females than in males [29]. This evidence also supports the above mentioned higher clinical impact of COPD in females, likely due to the effects of systemic inflammation which can affect biological pathways more heavily in a gender-dependent manner.

Despite digestive disorders appear clearly more frequent in females in absolute terms, only gall-bladder stones proved a clear gender-dependent prevalence in females, even if inflammatory gastric disorders seem to prevail in males. Moreover, only osteoporosis and spontaneous fractures represent a sort of females’ privilege, while arthrosis (and related disorders) represent a common characteristic of males with COPD. Independently of long-term corticosteroid use, anaemia occurrence, and possible-skeletal muscle dysfunction, the clear gender-dependency of these particular comorbidities can be likely suggested as mainly related to the hormonal disorders and the vitamin D deficiency which peculiarly characterize the second half of females’ life [28].

As in other previous studies, degenerative brain disorders together with cognitive disturbances and depression proved the most common neurological disorders related to COPD [7, 8, 30]. In particular, the occurrence of substantial limitations in cognition had been recently assessed by means of different psychometric instruments in COPD in proportion to the extent of chronic airway obstruction [31], and then it has been suggested as likely related to the extent of systemic inflammation. Furthermore, if the COPD males’ profile seems mainly characterized by the occurrence of neurological disorders (such as, dementia and limitations in cognition related to degenerative brain disorders), that of COPD females proves mostly characterized by the occurrence of peculiar psychological disorders, such as depression, also in agreement with a recent Italian survey [19]. Also in this case, sociologic, but also biologic (i.e. hormonal, vascular, etc.) determinants might contribute to this gender-dependent difference.

Finally, in the oncologic field, independently of those cancers which take their origin from gender-specific organs (such as, prostate in males; breast and the gynaecologic district in females), to point out that if the lung cancer ranked, as expected, at the first place in men, it, however, raised up to the second rank (only preceded by the thyroid cancer) in females, thus suggesting in this case its worrying progressive increase, almost independent of gender.

When the prevalence of comorbidities is investigated according to the different GOLD stages, they show a clear progressive increase from stage I to stage IV of COPD severity, except cardio-vascular and metabolic disorders which maintain this progression only up to the III GOLD stage, but show a dramatic drop of their prevalence just in the IV GOLD stage. If it is well accepted that systemic inflammation is of increasing extent during COPD progression, the most plausible hypothesis for explaining this strange inconsistency is that patients affected from most severe and most complicated cardio-vascular and/or metabolic disorders presumably have a higher mortality rate within the IV GOLD. This substantial mortality obviously leads to a selection of patients, and the final outcome (such as, the apparent drop in the prevalence of this kind of comorbidities in the extreme stage of COPD severity) is only mirroring a selection bias, and a misleading outcome. Actually, this epidemiologic feature should be instead regarded as a very severe outcome for these patients.

This study has some limitations. Firstly, it is a cross-sectional study and it does not provide any perspective information in the present version. Secondly, also specific information related to different phenotypes of COPD had not been provided with present data. Nevertheless, data collected in this first phase of the study are in agreement with, and confirm those of bigger studies carried out in different countries. The occurrence and the severity of comorbidities during the natural history of COPD further confirm their role in affecting the socio-economic impact, the quality of life, and mortality of COPD substantially.

## Conclusions

The gender-dependency of comorbidities in COPD was also proved: sometimes according to an expected evidence, but sometimes according to unexpected trends as in the case of lung cancer, which is suggesting the existence of a substantial epidemiological rearrangement in COPD-related disorders when compared to the corresponding status of a recent past.
